# Supplementary material for: The coilin N-terminus mediates multivalent interactions between coilin and Nopp140 to form and maintain Cajal bodies
Source: Nat Commun. 2022 Oct 12;13:6005. doi: 10.1038/s41467-022-33434-2 (PMC9556525; doi:10.1038/s41467-022-33434-2)
Supplement: Supplementary file 1 — Supplementary Information [file 41467_2022_33434_MOESM1_ESM.pdf]

SUPPLEMENTARY INFORMATION

The coilin N-terminus mediates multivalent interactions between coilin and Nopp140 to form and maintain Cajal bodies

Edward Courchaine*[1], Sara Gelles-Watnick*[1], Martin Machyna*[1], Korinna Straube[1], Sarah Sauyet[1], Jade Enright[1], and Karla M. Neugebauer[1]

[1]Department of Molecular Biophysics and Biochemistry, Yale University, New Haven

Corresponding Author: karla.neugebauer@yale.edu

*These authors contributed equally

Supplementary Figure 1. Properties of the coilin NTD *in vivo*. Related to Fig 1 (legend next page).

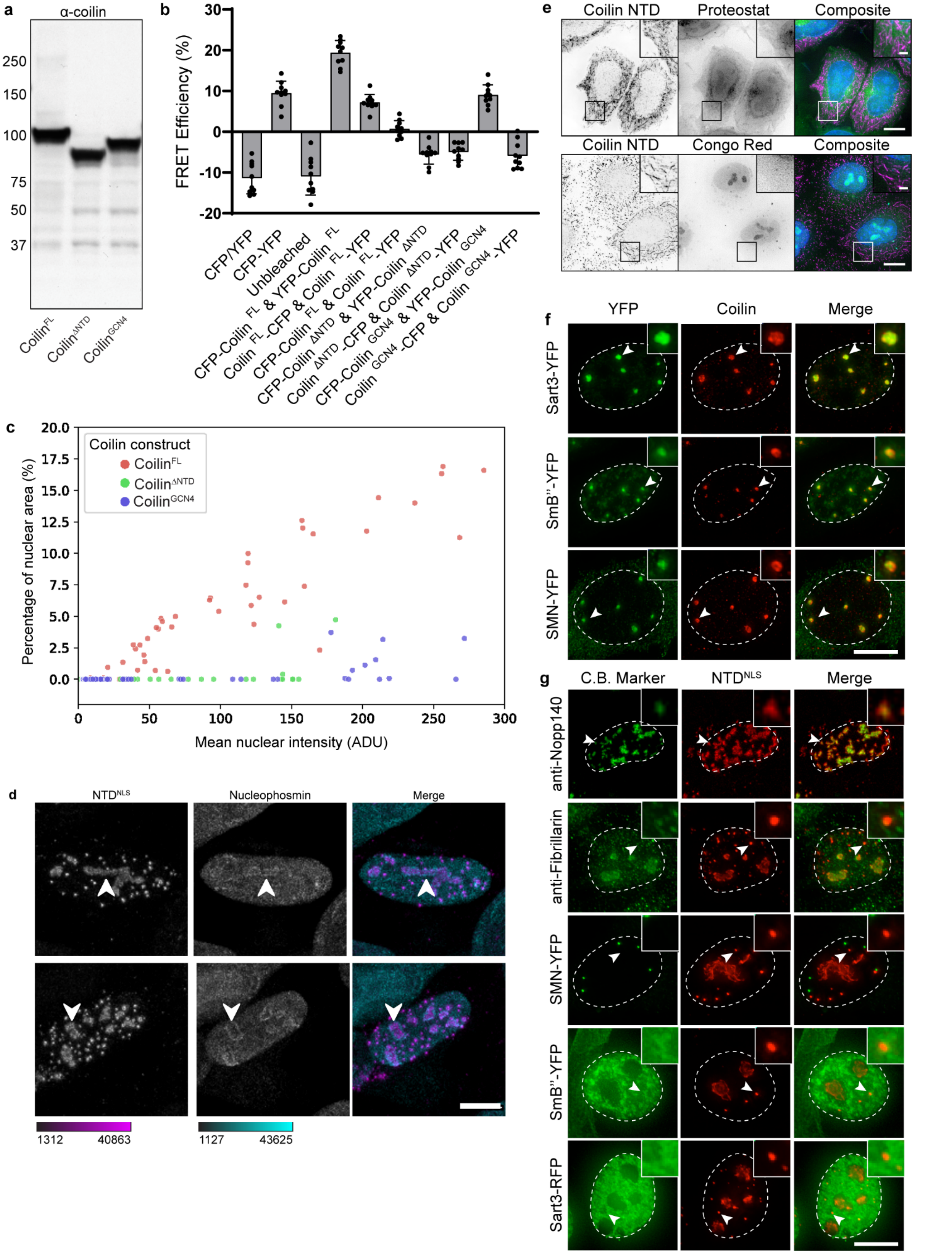

Supplementary Figure 1. Properties of the coilin NTD *in vivo*. Related to Fig 1.

A) Western blot of lysate from *coil* $^{-/-}$ MEFs expressing transiently transfected coilin constructs. The molecular weight markers are indicated to the left of the blot. **B)** FRET analysis performed in *coil* $^{-/-}$MEFs with fluorescent protein donors and acceptors included at both N and C termini. The first 3 determinations are controls: YFP an CFP expressed alone and mixed together (CFP/YFP) as a negative control, a fusion protein of CFP plus YFP (CFP-YFP) as a positive control, and a control for the effect of bleaching the fluorophores independent of the measured FRET interactions (unbleached), which yields a negative number. Note that subsequent negative values are due to photobleaching of the CFP donor. Next, coilin tagged at either the C-terminus or the N-terminus with CFP and YFP elicits FRET, whereas mixing the N- and C-termini labels does not produce FRET (previously shown in Stanek et. al. 2004); this robustly detect apparent FRET is abolished by deletion of the NTD, as indicated. In contrast, replacement of the NTD with GCN4 restores FRET when the fluorophores are placed on the N-terminus. Data represented as mean ± standard deviation (error bars). n = 12 cell measurements per condition. Black dots represent individual data points. **C)** Relative saturation concentration comparison of coilin constructs plotted as the percentage of nuclear area occupied by condensates over the mean fluorescence of coilin staining in each nucleus given in arbitrary digital units (ADU). Condensation was determined by a manual threshold of 20% of the detector dynamic range. Red dots, green dots, and blue dots represent full-length coilin, coilin$^{\Delta 97}$, and coilin^{GCN4}, respectively. **D)** Coilin NTD^{NLS} transiently transfected into *coil* $^{-/-}$MEFs. NTD^{NLS} was immunostained with anti-myc (magenta) and counterstained with anti-nucleophosmin (cyan). White arrows denote nucleoli, where NTD^{NLS} and nucleophosmin colocalize. NTD^{NLS} puncta do not colocalize with nucleophosmin. Color scale bars given in analog-digital units. Scale bar = 10 µm. Images acquired with Leica Sp8 laser scanning confocal microscope. This experiment was repeated independently two times with similar results. **E)** Coilin NTD expressed in HeLa cells and counterstained with dyes sensitive to amyloid cross-beta structures. Scale bar = 10 µm, Inset scale bar = 2 µm. **F)** YFP-tagged Cajal body markers co-transfected with Coilin^{FL} into *coil* $^{-/-}$MEFs. Coilin (red) is visualized by immunostaining. Arrowheads indicate example Cajal bodies enlarged in the inset. Scale bar = 10 µm. **G)** Primary antibody staining or transfection of YFP-tagged Cajal body markers in *coil* $^{-/-}$MEFs transfected with coilin NTD^{NLS}. Arrowheads indicate NTD bodies enlarged in the inset. Scale bar = 10 µm. All images acquired with DeltaVision. Source data are provided as a Source Data file.

Supplementary Figure 2. Nopp140 depletion disperses Cajal body components. Related to Fig 2.

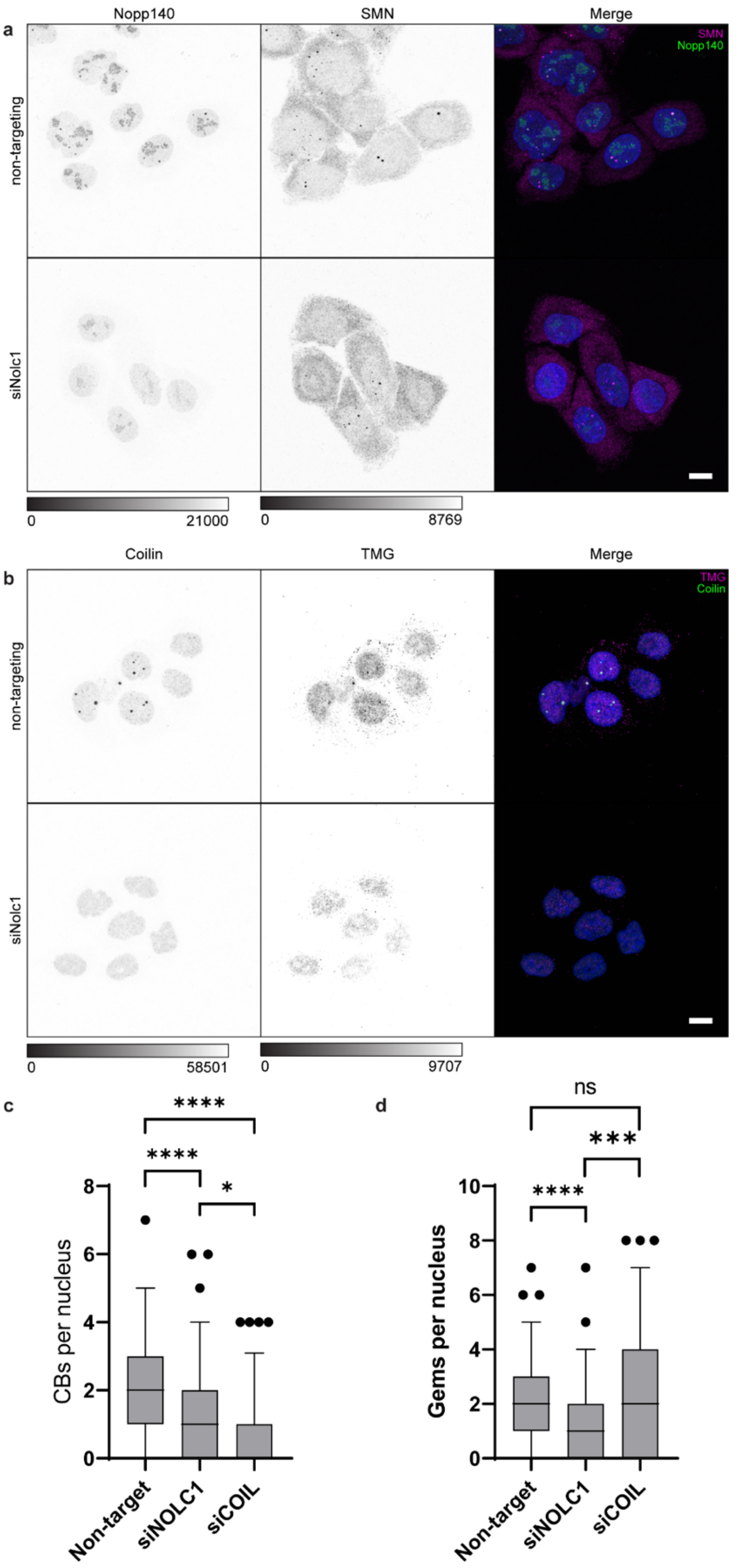

A) Representative images for cells undergoing transfection by a non-targeting oligo or si*Nolc1* oligo pool and labeled for Nopp140 (green) and SMN (magenta). DNA visualized by Hoechst (blue). Display levels noted by grayscale bars below. Scale bars = 10 µm. **B)** Representative images for cells undergoing transfection by a non-targeting oligo or si*Nolc1* oligo pool and labeled for Coilin (green) and trimethylguanosine (TMG; magenta). DNA visualized by Hoechst (blue). Display levels noted by grayscale bars below. Scale bars = 10 µm. C&D) Box plots generated from independent manual counting of microscopy data, indicating the number of coilin puncta **(C)** or SMN puncta **(D)** per nucleus in cells undergoing depletion. These manual data confirm the results obtained with automated counting presented in Fig 2. The center line represents the median. The box is drawn from the 25th to the 75th percentile. The whiskers are drawn from the 5th percentile to the 95th percentiles. Black dots represent individual data points. A two-sided Mann-Whitney test was performed. (C) n = 138 Non-target, 136 siNOLC1, 97 siCOIL cells quantified over 3 independent experiments. p = <0.0001 Non-target vs. siNOLC1, <0.0001 Non-target vs. siCOIL, 0.048 siNOLC1 vs. siCOIL. (D) n = 137 Non-target, 139 siNOLC1, 104 siCOIL cells quantified over 3 independent experiments. p = <0.0001 Non-target vs. siNOLC1, 0.41 Non-target vs. siCOIL, 0.0002 siNOLC1 vs. siCOIL.(*) indicates $p < 0.05$, (***) indicates $p < 0.001$, and (****) indicates $p < 0.0001$ for Mann Whitney test. Source data are provided as a Source Data file.

Supplementary Figure 3. Negative and positive controls for optodroplet assay. Related to Fig 3.

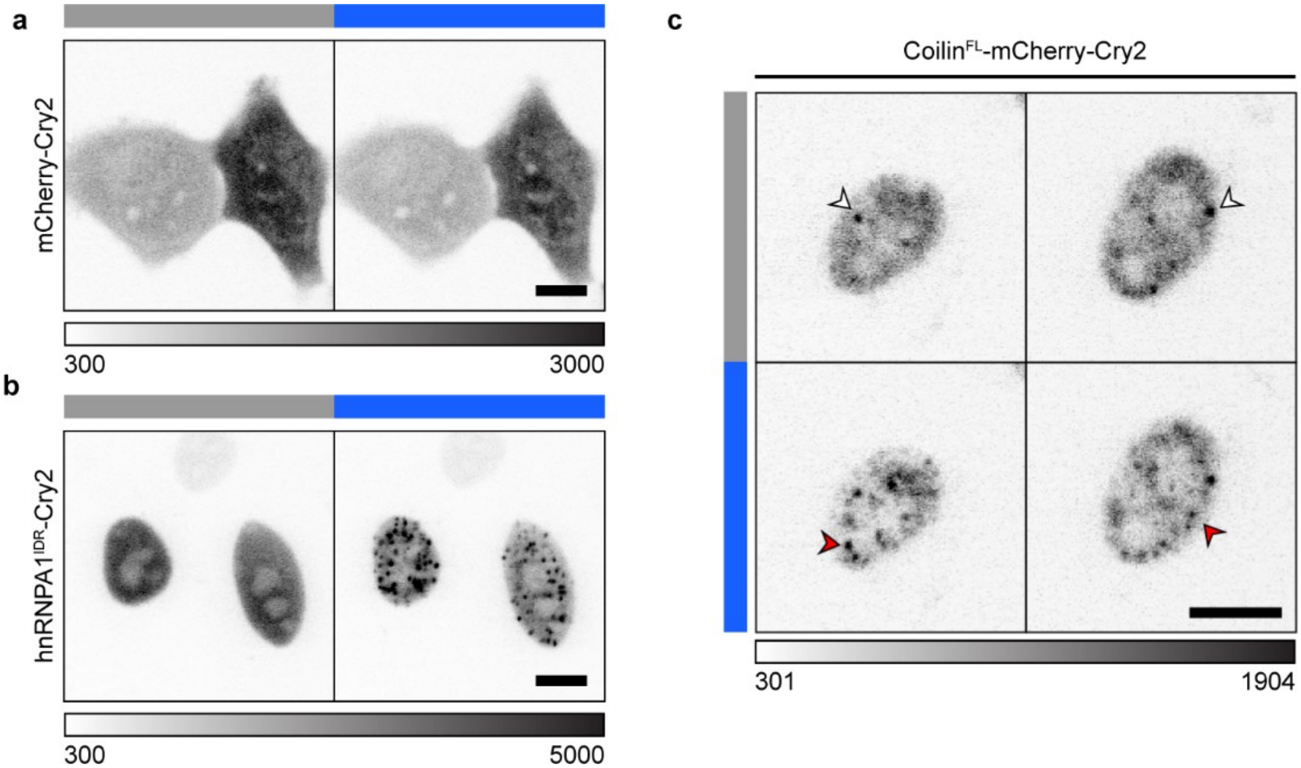

A) Live-cell imaging of NIH-3T3 cells expressing mCherry-Cry2, **B)** the hnRNPA1 intrinsically disordered region, and **C)** full length coilin-Cry2. Blue bar indicates 180 s activation with blue light. Grayscale bars given in analog-digital units. Scale bars = 10 µm. White arrows in panel C indicate foci before blue light activation, red arrows indicate foci formed upon blue light activation.

Supplementary Figure 4. Alignment of vertebrate coilin sequences indicates highly conserved residues. Related to Fig 4.

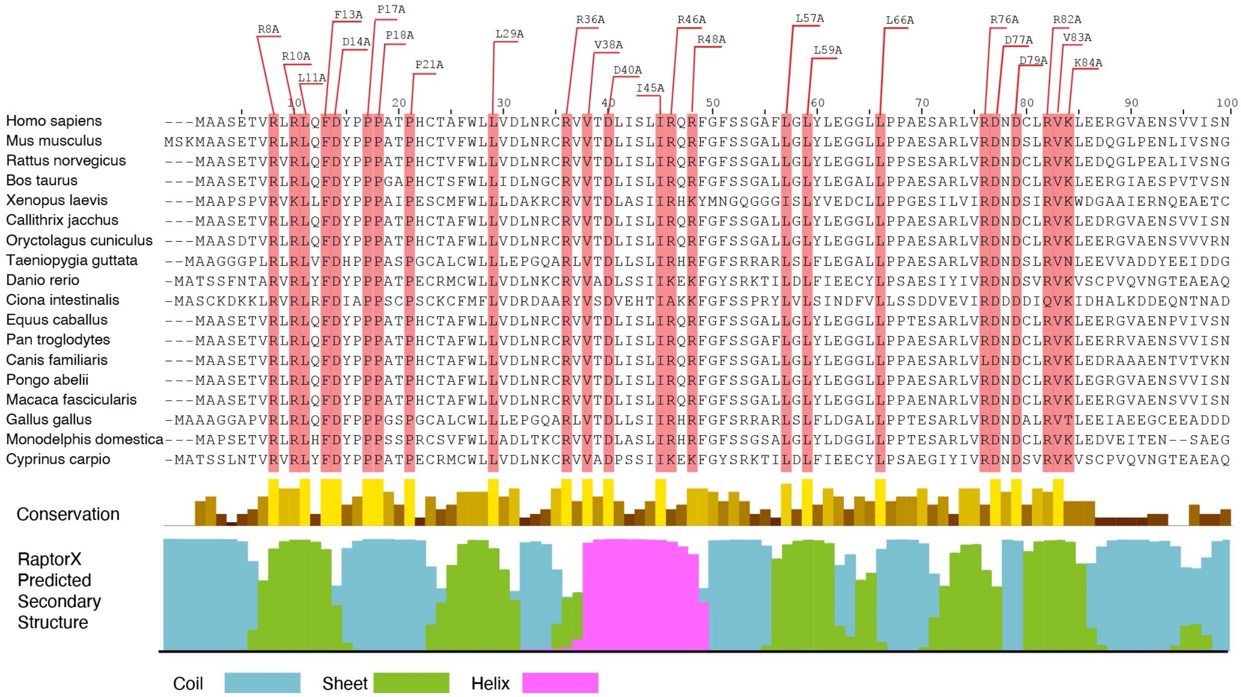

Multiple sequence alignment of vertebrate coilin orthologs, cropped to the first one hundred amino acids. Conservation track is computed in Clustal Omega and reveals highly conserved residues selected for alanine scanning mutagenesis, highlighted in red. RaptorX secondary structure predictions indicate possible regions of beta-strands (green) or alpha-helix (magenta).

Supplementary Figure 5. Mutation of conserved residues reveals key interaction sites of the coilin NTD. Related to Fig 4 (legend next page).

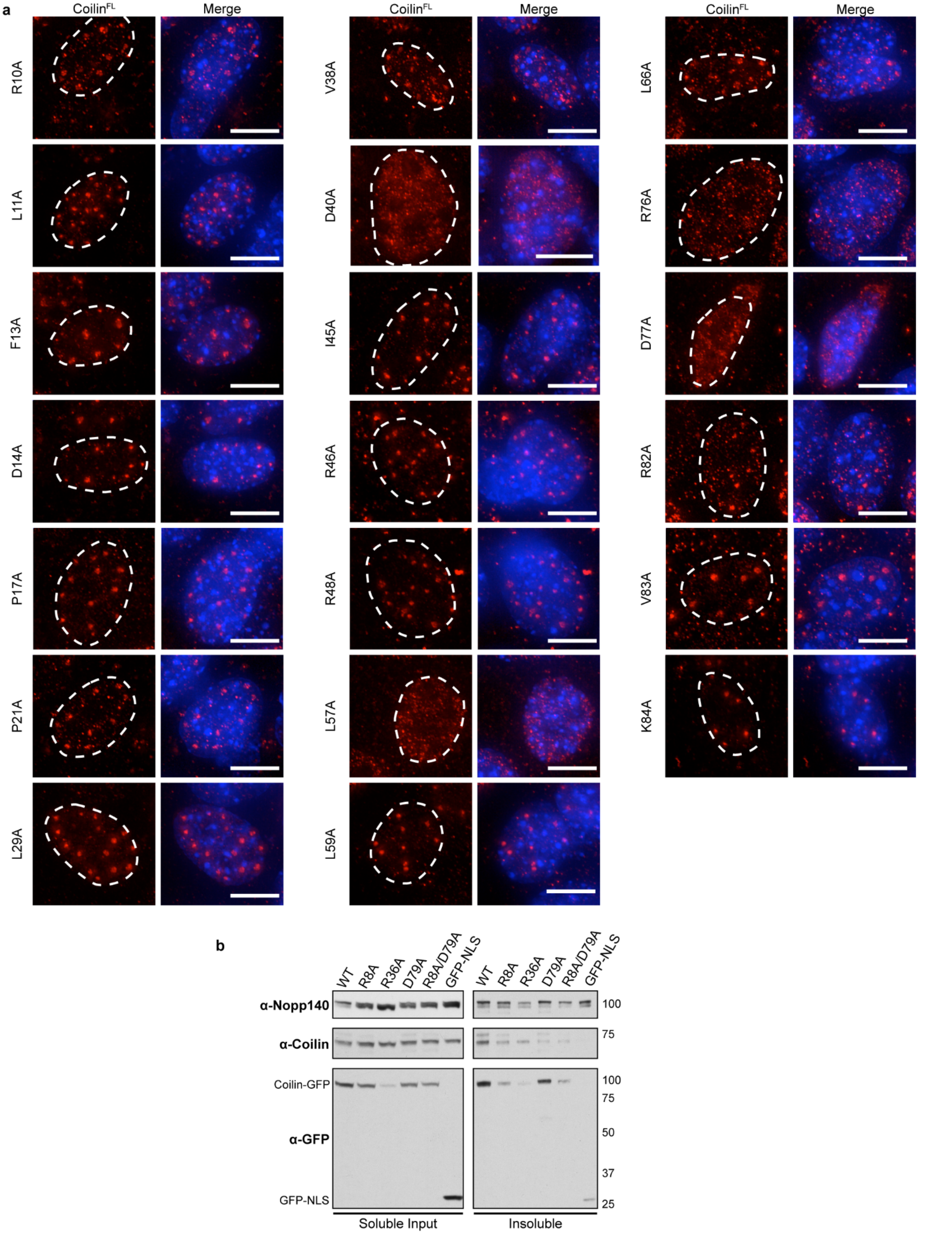

Supplementary Figure 5. Mutation of conserved residues reveals key interaction sites of the coilin NTD. Related to Fig 4.

A) Coilin (red) transfected into *coil* $^{-/-}$MEFs and visualized by immunofluorescence. Mutations correspond to the red highlighted residues in Figure S4, with numbering according to the human coilin sequence. Blue indicates nuclear staining. Images acquired with DeltaVision. Scale bars = 10 µm. **B)** Soluble input and insoluble pellet from the immunoprecipitation experiment shown in Figure 3. Bolded text indicates the antibodies used. Lysates were produced from HeLa cells. Molecular weight markers are indicated to the right of the blots. Source data are provided as a Source Data file.

Supplementary Figure 6. Effect of NTD point mutations on cellular properties. Related to Fig 5.

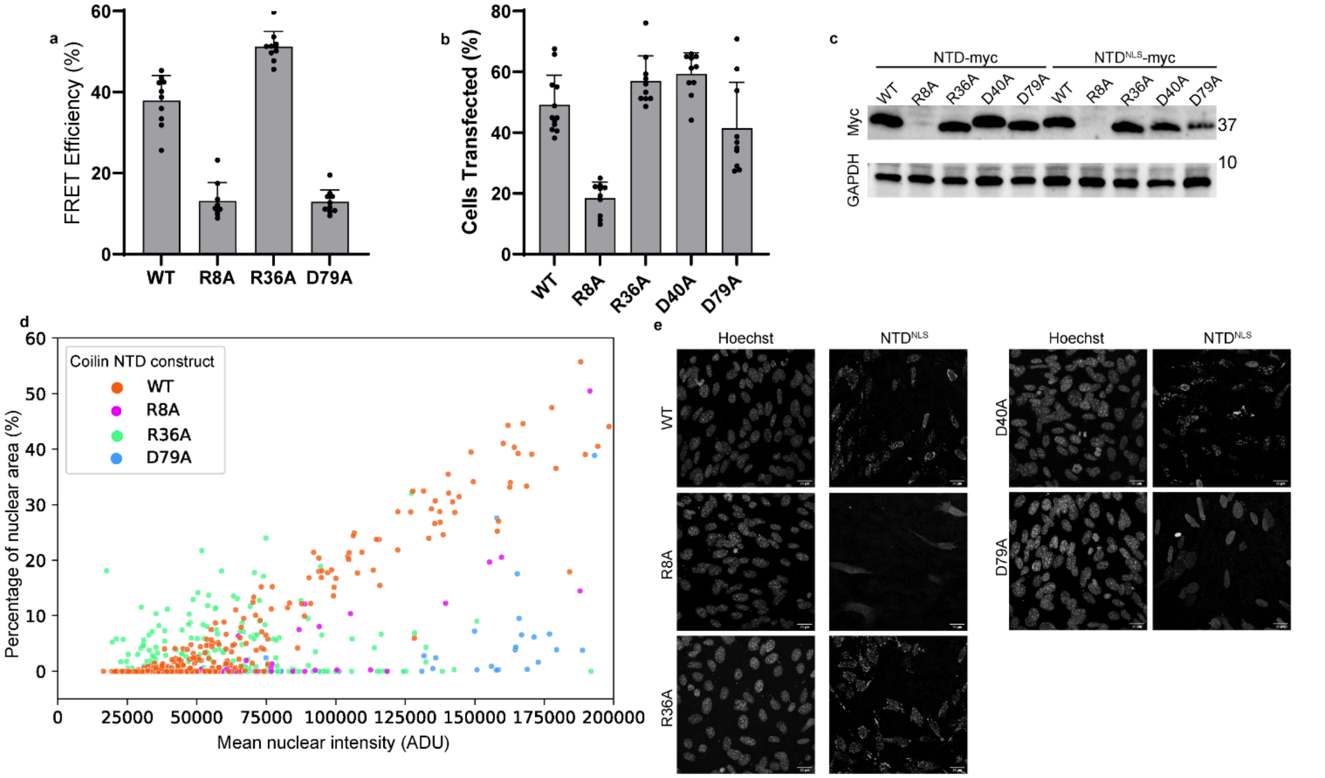

A) FRET analysis of CFP- and YFP-labeled coilin NTD constructs, co-transfected into *coil* $^{-/-}$MEFs. Data are represented as mean values ± standard deviation (error bars). n = 10 cells measured for each construct. Black dots indicate individual data points. See Methods and Fig S1b for further details of the assay. **B)** WT and mutant Coilin NTD^{NLS} were transiently transfected into *coil* $^{-/-}$MEFs. NTD^{NLS} constructs were immunostained with anti-myc and Hoechst. For each field of view, number of transfected cells (positive for anti-myc) and total number of cells (positive for Hoechst) were counted by eye. The transfection percentage was calculated accordingly for each construct and mean (bar height) ± standard deviation (error bar) is shown for 10 fields of view over 3 independent experiments. Black dots indicate individual data points. **C)** Western blot performed on cells transiently transfected with NTD-myc and NTD^{NLS}-myc WT and mutant constructs. Stained for myc and GAPDH was used as a loading control. This blot was not repeated; it suggested that our R8A construct is poorly transfected, which was directly and rigorously tested in Figs D and E, as follows: **D)** Relative saturation concentration comparison of coilin NTD-NLS constructs plotted as the percentage of nuclear area occupied by condensates over the mean fluorescence of coilin staining in each nucleus given in arbitrary digital units (ADU). Condensation was determined by a manual threshold of 40% of the detector dynamic range. Orange dots, pink dots, green dots, and blue dots represent NTD-NLS WT, R8A, R36A, and D79A, respectively. **E)** WT and mutant Coilin NTD^{NLS} constructs were transiently transfected into *coil* $^{-/-}$MEFs. NTD^{NLS} constructs were immunostained with anti-myc and Hoechst. The transfection efficiency from (B) was calculated from fields of view such as these. Source data are provided as a Source Data file.

Supplementary Figure 7. NTD double point mutants do not interact with each other or endogenous coilin. Related to Fig 6.

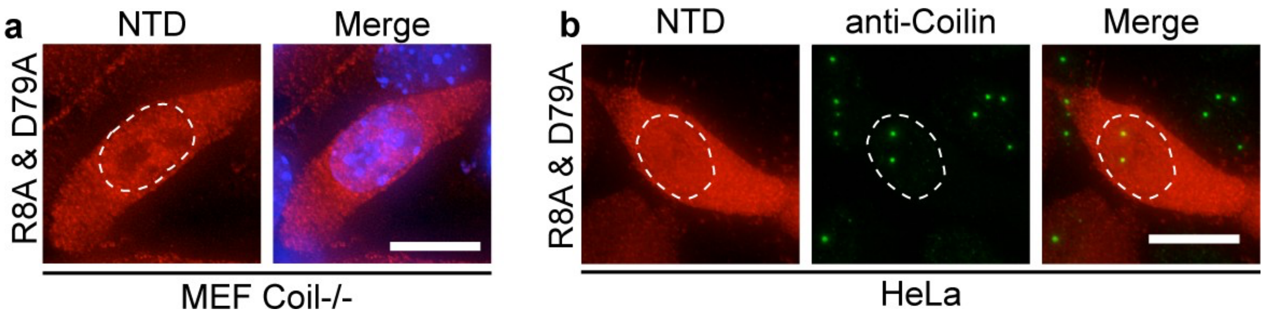

A) Coilin NTD (red) bearing both R8A and D79A mutations transfected into *coil* $^{-/-}$MEFs. Nuclei boundaries indicated with white dashed line. Scale bar = 10 µm. **B)** Coilin NTD (red) bearing both R8A and D79A mutations transfected into HeLa and counterstained for coilin (green). Nuclei boundaries indicated with white dashed line. Images acquired with DeltaVision. Scale bar, 10 µm.
