# Supplementary material for: The coilin N-terminus mediates multivalent interactions between coilin and Nopp140 to form and maintain Cajal bodies
Source: Nat Commun. 2022 Oct 12;13:6005. doi: 10.1038/s41467-022-33434-2 (PMC9556525; doi:10.1038/s41467-022-33434-2)
Supplement: Supplementary file 3 — Reporting Summary [file 41467_2022_33434_MOESM3_ESM.pdf]

nature portfolio

Corresponding author(s): Karla Neugebauer

Last updated by author(s): 2022/08/26

Reporting Summary

Nature Portfolio wishes to improve the reproducibility of the work that we publish. This form provides structure for consistency and transparency in reporting. For further information on Nature Portfolio policies, see our Editorial Policies and the Editorial Policy Checklist.

Statistics

For all statistical analyses, confirm that the following items are present in the figure legend, table legend, main text, or Methods section.

n/a	Confirmed	
☐	☒	The exact sample size (n) for each experimental group/condition, given as a discrete number and unit of measurement
☐	☒	A statement on whether measurements were taken from distinct samples or whether the same sample was measured repeatedly
☐	☒	The statistical test(s) used AND whether they are one- or two-sided<br>*Only common tests should be described solely by name; describe more complex techniques in the Methods section.*
☒	☐	A description of all covariates tested
☒	☐	A description of any assumptions or corrections, such as tests of normality and adjustment for multiple comparisons
☒	☐	A full description of the statistical parameters including central tendency (e.g. means) or other basic estimates (e.g. regression coefficient) AND variation (e.g. standard deviation) or associated estimates of uncertainty (e.g. confidence intervals)
☐	☒	For null hypothesis testing, the test statistic (e.g. F, t, r) with confidence intervals, effect sizes, degrees of freedom and P value noted<br>*Give P values as exact values whenever suitable.*
☒	☐	For Bayesian analysis, information on the choice of priors and Markov chain Monte Carlo settings
☒	☐	For hierarchical and complex designs, identification of the appropriate level for tests and full reporting of outcomes
☒	☐	Estimates of effect sizes (e.g. Cohen's d, Pearson's r), indicating how they were calculated

Our web collection on statistics for biologists contains articles on many of the points above.

Software and code

Policy information about availability of computer code

Data collection	LASX, Deltavision software, PrairieView
Data analysis	RaptorX, GraphPadPrism v9.4.1, Clustal Omega, ImageJ (FIJI), Cell Profiler 4.2.171

For manuscripts utilizing custom algorithms or software that are central to the research but not yet described in published literature, software must be made available to editors and reviewers. We strongly encourage code deposition in a community repository (e.g. GitHub). See the Nature Portfolio guidelines for submitting code & software for further information.

Data

Policy information about availability of data

All manuscripts must include a data availability statement. This statement should provide the following information, where applicable:

- Accession codes, unique identifiers, or web links for publicly available datasets
- A description of any restrictions on data availability
- For clinical datasets or third party data, please ensure that the statement adheres to our policy

This study did not generate large datasets or computer code. There are no restrictions on the availability of any of our data.

Field-specific reporting

Please select the one below that is the best fit for your research. If you are not sure, read the appropriate sections before making your selection.

☒ Life sciences ☐ Behavioural & social sciences ☐ Ecological, evolutionary & environmental sciences

For a reference copy of the document with all sections, see nature.com/documents/nr-reporting-summary-flat.pdf

Life sciences study design

All studies must disclose on these points even when the disclosure is negative.

Sample size	Sample sizes for tests of molecular function using fluorescence microscopy: In Fig 2, we counted 30-50 cells for each condition and for each of three biological replicates and then pooled. The Mann Whitney test was used to derive p-values shown. In the other figures with fluorescence analysis of phenotypes, 300 cells per condition were categorized and tabulated as the fraction of cells with any particular morphology. P-values were not calculated because the data were yes/no (like, was the object round or straight?) and not parametrized. These sample sizes are typical for these assays when performed by our and other labs in the field.
Data exclusions	no data were excluded.
Replication	In all experiments, 3 replicates were performed. All replicates that passed quality control standards for technical performance (e.g. the western blot had efficient transfer of the protein) were in agreement with the other replicates. We have made a statement in the methods that all figure legends state if the number of replicates deviated from 3. The effects of mutations were highly reproducible. Standard error of the mean is shown.
Randomization	The slides were randomized so that the order of the slides did not contain information about sample identity (see below).
Blinding	Quantification of visually evaluated data (see above) was accomplished double blind by marking the glass coverslips with a code and handing a separate, trained individuals with slides they could not recognize vis a vis the experimental treatment.

Reporting for specific materials, systems and methods

We require information from authors about some types of materials, experimental systems and methods used in many studies. Here, indicate whether each material, system or method listed is relevant to your study. If you are not sure if a list item applies to your research, read the appropriate section before selecting a response.

Materials & experimental systems

n/a	Involved in the study	
☐	☒	Antibodies
☐	☒	Eukaryotic cell lines
☒	☐	Palaeontology and archaeology
☒	☐	Animals and other organisms
☒	☐	Human research participants
☒	☐	Clinical data
☒	☐	Dual use research of concern

Methods

n/a	Involved in the study	
☒	☐	ChIP-seq
☒	☐	Flow cytometry
☒	☐	MRI-based neuroimaging

Antibodies

Antibodies used	SMN (2B1), ab5831, Abcam<br>Coilin, ab210785, Abcam<br>Myc (9E10), sc-40, Santa Cruz<br>Nolc1, NBP1-22982, NovusBio<br>GFP, A11122, Invitrogen<br>Cy™5 AffiniPure Goat Anti-Rabbit IgG, 111-175-144, Jackson ImmunoResearch<br>Alexa Fluor 488-conjugated AffiniPure Donkey Anti-Rabbit IgG, 711-545-152, Jackson ImmunoResearch<br>Alexa Fluor 488-conjugated AffiniPure Donkey Anti-Mouse IgG, 715-545-150, Jackson ImmunoResearch<br>Anti-rabbit IgG Horseradish Peroxidase-Linked Species-Specific Whole Antibody, NA934, GE HealthCare
Validation	All of the above-listed antibodies were validated by Western blotting and/or immunostaining, including titration of the concentration with each new batch. Human HeLa cells and mouse MEF -/- cells were used for validation. These experiments are data-not-shown, but the concentrations of antibodies used and given in the main and supplementary figures/figure legends reflect these determinations.

Eukaryotic cell lines

Policy information about cell lines

Cell line source(s)	Mouse coil -/-MEFs were a gift of Greg Matera. HeLa cells are from the Kyoto linage (RRID: CVCL_1922) and were obtained from the Genome Engineering Facility at MPI-CBG in Dresden Germany; the cell line is fully described here https://pubmed.ncbi.nlm.nih.gov/30778230/. Cry2 optodroplet experiments were performed in NIH-3T3 cells obtained from ATCC (#CRL-1658). 293FT cells were used as a lentivirus packaging line only and were obtained from Thermo (#R70007).
Authentication	NIH 3T3 cells were obtained from ATCC in 2019 for the purpose of another study (https://pubmed.ncbi.nlm.nih.gov/34115980/); because this is recent, we did not authenticate further. Genetic deletion in the Coil-/-MEFs was validated by sequencing and western blotting. HeLa-Kyoto cells are described above; They have a recognizable cuboidal shape that is favorable for microscopy. It is impossible to confuse them with other HeLa cells in the lab.
Mycoplasma contamination	All cells are periodically (appx every 6 months) tested for mycoplasma. Therefore, we consider the lines mycoplasma negative.
Commonly misidentified lines (See ICLAC register)	No commonly misidentified cell lines were used in this study.
